# Monocytohigh-density lipoprotein ratio has a high predictive value for the diagnosis of multiple system atrophy and the differentiation from Parkinson’s disease

**DOI:** 10.3389/fnagi.2022.1035437

**Published:** 2022-10-13

**Authors:** Lijuan Jiang, Zhen Zhong, Juan Huang, Hetao Bian, Wei Huang

**Affiliations:** ^1^Department of Neurology, The Second Affiliated Hospital of Nanchang University, Nanchang, China; ^2^Department of Neurology, Beijing Institute for Brain Disorders, Capital Medical University, Beijing, China

**Keywords:** multiple system atrophy, Parkinson’s disease, monocyte to high-density lipoprotein ratio, neutrophil to lymphocyte ratio, red cell distribution width to platelet ratio, peripheral inflammation

## Abstract

**Background and purpose:**

Inflammation is closely related to the pathogenesis of multiple system atrophy (MSA). As markers of inflammation, the monocyte to high-density lipoprotein ratio (MHR), neutrophil to lymphocyte ratio (NLR), and red cell distribution width to platelet ratio (RPR) have been proven to be associated with a large variety of diseases. The aim of this study was to explore the association between inflammatory markers (MHR, NLR, and RPR) and MSA, and the difference between MSA and Parkinson’s disease (PD) was further compared by these inflammatory markers.

**Materials and methods:**

This study was divided into three groups: 47 patients with MSA, 125 patients with PD, and 124 healthy controls. The corresponding laboratory indicators of subjects were collected and analyzed to obtain MHR, NLR, and RPR values.

**Results:**

Compared with healthy controls, the MHR, NLR, and RPR were higher in the MSA group (*P* < 0.05), and the MHR was higher in the MSA group than in the PD group (*P* < 0.001). Multivariate logistic regression analysis showed that MHR*10 (corrected OR = 1.312, 95% CI 1.093–1.575) and RPR*100 (corrected OR = 1.262, 95% CI 1.055–1.509) were positively correlated with the risk of MSA. The receiver operating characteristic (ROC) curve indicated that the areas under the curve (AUCs) of the MHR, NLR, and RPR for predicting MSA were 0.651 (95% CI 0.562–0.74; *P* < 0.05), 0.6 (95% CI 0.501–0.699; *P* < 0.05), and 0.612 (95% CI 0.516–0.708; *P* < 0.05), respectively. The AUC of MSA and PD predicted by the MHR was 0.727 (*P* < 0.001). When the cut-off value was 0.38, the sensitivity and specificity were 60 and 77%, respectively. Spearman correlation analysis showed that the MHR was significantly and positively correlated with the course of MSA cerebellar type (MSA-C) patients.

**Conclusion:**

There may be peripheral inflammation in patients with MSA. Compared with NLR and RPR, MHR has higher predictive value for the diagnosis and differential diagnosis of MSA.

## Introduction

Known as multiple system atrophy (MSA), this neurodegenerative condition affects multiple systems. MSA is a disease that causes Parkinsonian symptoms as well as cerebellar and autonomic dysfunction, with various clinical manifestations. MSA falls into two categories: multiple system atrophy-parkinsonism (MSA-P) and multiple system atrophy-cerebellar ataxia (MSA-C) (Fanciulli and Wenning, 2015). Some scholars have found that the incidence rate of MSA is 0.11/1,00,000 person-years, which is relatively rare (Winter et al., 2010). The pathology of MSA involves deposition of α-synuclein (α-synuclein). Parkinson’s disease (PD) is another neurodegenerative disease, and its pathological changes are mainly related to the deposition of α-synuclein. Both PD and MSA belong to α-synucleinopathy. At present, a study has confirmed the correlation between PD and peripheral inflammation. When the level of peripheral inflammation increases, microglia may sense changes in the peripheral environment, and release reactive oxygen species and cytokines, activating astrocytes, oligodendrocytes, and vascular endothelial cells to further release inflammatory factors, accelerating the aggregation of α-synuclein and ultimately leading to neuronal damage (Subeta et al., 2021).

The pathogenesis of MSA is unclear, and neuroinflammation may be one of them. Harms et al. (2021) confirmed that elevated proinflammatory factors and T lymphocyte invasion were found in the cerebrospinal fluid of MSA patients and animal models. Williams et al. (2020) observed the proliferation and activation of inflammatory microglia and peripheral monocytes in the central nervous system (CNS) in MSA mouse models. The above studies mainly focused on the CNS, while there are few studies on peripheral inflammation in MSA, and there are no consistent conclusion. The neutrophil to lymphocyte ratio (NLR), monocyte to high-density lipoprotein ratio (MHR), and red cell distribution width to platelet ratio (RPR) are novel indicators of peripheral inflammation. For the past few years, studies have indicated that they are involved in tumors, cardiovascular diseases, and neurodegenerative diseases such as PD and progressive supranuclear palsy (PSP) (Inci et al., 2020; Li et al., 2021; Liu et al., 2021). Inflammation is involved in the pathogenesis of MSA. Additionally, both MSA and PD belong to α-synucleinopathy, showing similar pathological changes. Is there a similar relationship between MSA and peripheral inflammation? This study aims to explore the relationship between MSA and peripheral inflammation from the perspective of novel indicators of peripheral inflammation. As MSA only presents with Parkinsonian-like symptoms in the early stage, it is difficult to distinguish from PD. To provide clinical clues between MSA and PD for early identification, we further analyzed the differences in novel inflammatory indicators.

## Materials and methods

We collected the data of 172 patients hospitalized in the Department of Neurology of the Second Affiliated Hospital of Nanchang University from January 2016 to May 2022, including 47 patients with MSA and 125 patients with PD. Based on the Consensus of the American Academy of Neurology, the diagnosis was possible MSA (Gilman et al., 2008). MDS clinical diagnostic criteria were adopted to diagnose PD (Postuma et al., 2015). A total of 124 age- and sex-matched healthy patients were selected as the control group. Patients with the following diseases were excluded: (1) neurodegenerative diseases such as progressive supranuclear palsy, corticobasal degeneration, Lewy body dementia, front temporal dementia, and Alzheimer’s disease; (2) diabetes, hypothyroidism, tumors, and autoimmune diseases; (3) infection; (4) acute stroke; (5) liver or renal failure; and (6) use of hypolipidaemic drugs.

This study was a retrospective study and was approved by the Ethics Committee of the Second Affiliated Hospital of Nanchang University.

General information of all patients was recorded, including age, gender, disease duration, past medical history, tobacco and alcohol use and medication history, and family history. NLR, MHR, and RPR values were calculated by recording neutrophils, lymphocytes, monocytes, red cell distribution width, platelets (PLTs), and high-density lipoprotein (HDL) values. The units of neutrophils, monocytes, lymphocytes, and PLTs were all ×10^9^/L. The reference ranges of red cell distribution width (RDW-CV) and HDL were 10.9–15.4% and 1.16–1.42 mmol/L, respectively. All detection indicators were measured by an automatic hematology analyzer (Sysmex XN-20A1, Kobe, Japan) and automatic biochemical analyzer (Beckman AU5800, Brea, CA, USA).

SPSS software (SPSS version 26.0; SPSS Inc., Chicago, IL, USA) was used for statistical analysis. The Kolmogorov–Smirnov test was used for normality testing. The measurement data conforming to the normality test were represented as the mean ± standard deviation (mean ± SD), and a *t*-test was adopted. When the measurement data did not conform to normality, the Mann–Whitney U test was used and data were expressed as the median (quartile) [M (P25, p75)]. The counting data were compared between groups by Pearson’s chi-square test and expressed as *n* (%). Binary logistic regression was established by the backward LR method to analyze the relationship between novel inflammatory factors (NLR, MHR*10, and RPR*100) and the risk of MSA. The variables in univariate analysis (*P* < 0.1) were incorporated into the multifactor logistic regression analysis. To evaluate the predictive value of NLR, MHR, and RPR for MSA and PD, receiver operating characteristic (ROC) curves were plotted. Finally, Spearman’s correlation test was conducted to analyze the correlation between NLR, MHR, RPR and course of disease in patients with different types of MSA. In all of the above analyses, *P* < 0.05 was recognized as statistically significant.

## Results

### Comparison of baseline data and laboratory indicators among the three groups

In the MSA group, there were 47 cases, including 30 males (63.8%), with an age of 61 years (55–66). Among 125 PD patients, 72 (57.6%) were males, with an age of 63 years (56–68). There were 124 healthy controls, including 60 males (48.4%), with an age of 58 years (52–67.75). There were no significant differences in age, sex, smoking, drinking or hypertension among the three groups (*P* > 0.05). Compared with the HC group, the MHR, NLR, RPR, and monocytes of the MSA group were higher, the distribution width of erythrocytes and HDL were lower, and the differences were statistically significant. Compared with the HC group, the RPR and red blood cell distribution width (RDW) were higher in the PD group (*P* < 0.05), and the neutrophils, monocytes, lymphocytes, PLTs, and MHR were lower (*P* < 0.05). The MSA group had higher levels of MHR, neutrophils, and monocytes and lower levels of HDL than the PD group (*P* < 0.05). The results are presented in Table 1.

**TABLE 1:** Comparison of baseline data and laboratory indicators among the three groups.

	MSA (*n* = 47)	PD (*n* = 125)	HC (*n* = 124)	*Z*1/χ1/*T*1	*P*1	*Z*2/χ2/*T*2	*P*2	*Z*3/χ3/*T*3	*P*3
Age (years)	61 (55–66)	63 (56–68)	58 (52–67.75)	−0.28	0.779	−1.907	0.057	−1.362	0.173
Sex (male, %)	30 (63.8%)	72 (57.6%)	60 (48.4%)	3.26	0.071	2.121	0.145	0.549	0.459
Smoking (%)	5 (10.6%)	10 (8%)	12 (9.7%)	0.035	0.851	0.271	0.641	0.299	0.585
Drinking (%)	7 (14.9%)	12 (9.6%)	8 (6.5%)	3.035	0.081	0.835	0.361	0.974	0.324
Hypertension (%)	12 (25.5%)	19 (15.2%)	26 (21%)	0.411	0.522	1.399	0.237	2.468	0.116
Hyperlipidemia (%)	8 (17%)	19 (15.2%)	22 (17.7%)	0.12	0.912	0.292	0.589	0.086	0.77
CHD (%)	1 (2.1%)	2 (1.6%)	0 (0%)	/	0.275	/	0.251	0	1
Family history (%)	1 (2.1%)	2 (1.6%)	1 (0.8%)	0	1	0	1	0	1
Antiplatelet drugs (%)	3 (6.4%)	8 (6.4%)	13 (10.5%)	0.279	0.598	1.344	0.246	0	1
Monocytes (×10^9^/L)	0.45 ± 0.134	0.34 ± 0.133	0.39 ± 0.127	2.54	0.012	−2.847	0.005	4.548	<0.001
HDL-C (×10^9^/L)	1.12 ± 0.325	1.25 ± 0.309	1.29 ± 0.386	−2.615	0.01	−0.884	0.378	−2.367	0.019
Neutrophils (×10^9^/L)	3.84 (2.81–4.66)	3.16 (2.575–3.84)	3.48 (2.785–4.18)	−1.467	0.142	−2.067	0.039	−2.771	0.006
Lymphocytes (× 10^9^/L)	1.68 ± 0.549	1.58 ± 0.481	1.76 ± 0.491	−0.866	0.388	−2.847	0.004	1.17	0.244
RDW-SD (cv, %)	12.9 (12.5–13.6)	13.0 (12.45–13.4)	12.6 (12.1–13.1)	−2.559	0.011	−3.421	0.001	−0.143	0.886
Platelets (× 10^9^/L)	197.49 ± 56.617	194 (169–230)	212.48 ± 55.77	−1.562	0.12	−2.12	0.034	−0.148	0.883
MHR	0.41 (0.28–0.56)	0.27 (0.19–0.37)	0.29 (0.21–0.45)	−3.045	0.002	−2.078	0.038	−4.582	<0.001
NLR	2.22 (1.62–3.27)	1.96 (1.56–2.75)	1.95 (1.58–2.61)	−2.007	0.045	−0.582	0.561	−1.328	0.184
RPR	0.07 (0.06–0.09)	0.07 (0.06–0.08)	0.06 (0.05–0.07)	−2.261	0.024	−2.703	0.007	−0.359	0.72
Parkinson’s drugs									
Compound levodopa (%)	18 (38.3%)	119 (96.7%)	/	/	/	/	/	74.266	<0.001
DR agonist (%)	4 (8.5%)	80 (64.0%)	/	/	/	/	/	39.9	<0.001
MAO-B inhibitors (%)	3 (6.4%)	17 (13.6%)	/	/	/	/	/	1.1	0.294
COMT inhibitors (%)	0 (0%)	6 (3.5%)	/	/	/	/	/	/	0.142
Anticholinergic drugs (%)	1 (2.1%)	18 (14.4%)	/	/	/	/	/	4.061	0.044
Amantadine (%)	1 (2.1%)	11 (8.8%)	/	/	/	/	/	1.428	0.232

MSA, multiple system atrophy; PD, Parkinson’s disease; HC, healthy controls; CHD, coronary heart disease; HDL-C, high-density lipoprotein cholesterol; RDW-SD, red cell distribution width; MHR, monocyte to HDL ratio; NLR, neutrophil to lymphocyte ratio; RPR, red cell distribution width to platelet ratio. 1, MSA vs. HC; 2, PD vs. HC; 3, MSA vs. PD.

### Risk factor analysis for multiple system atrophy

Multivariate logistic regression analysis showed that MHR*10 (corrected OR = 1.312, 95% CI 1.093–1.575) and RPR*100 (corrected OR = 1.262, 95% CI 1.055–1.509) were positively correlated with the risk of MSA, as shown in Table 2.

**TABLE 2:** Univariate and multivariate logistic regression analysis of multiple system atrophy (MSA) risk factors.

	Univariate logistics regression analysis		Multivariate logistics regression analysis	
	Crude OR (95% CI)	*P*	Adjusted OR (95% CI)	*P*
NLR	1.184 (0.907–1.547)	0.215	–	–
MHR*10	1.284 (1.078–1.529)	0.005	1.313 (1.093–1.575)	0.004
RPR*100	1.232 (1.04–1.459)	0.016	1.262 (1.055–1.509)	0.011

Since the values of MHR and RPR are a*10^–1^ and b*10^–2^, respectively (0 < a < 10, 0 < b < 10). Carry out logistic regression analysis. When the independent variable is increased by 1 unit, the MHR value is equivalent to expanding by 10 times, and the RPR value is equivalent to expanding by 100 times. The obtained OR value will be amplified. Therefore, MHR*10 and RPR*100 conversion are made.

### Receiver operating characteristic curve analysis

**FIGURE 1:**
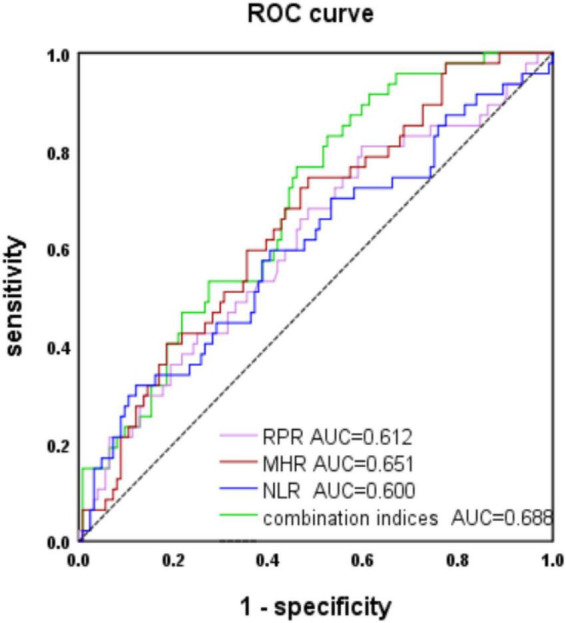
Receiver operating characteristic (ROC) curve analysis of monocyte to high-density lipoprotein ratio (MHR), neutrophil to lymphocyte ratio (NLR), and red cell distribution width to platelet ratio (RPR) to predict multiple system atrophy (MSA).

We evaluated the predictive value of the MHR, NLR, and RPR for MSA, and the AUCs of the MHR, NLR, and RPR were 0.651, 0.6, and 0.631, respectively (*P* < 0.05) (Figure 1). The cut-off value for the MHR was 0.3, for which the sensitivity for the prediction of MSA was 75% and the specificity was 52% (95% CI 0.562–0.74).

The AUC of MHR combined with NLR and RPR was 0.688 (*P* < 0.001). When the cut-off value was 0.22, it predicted MSA with 77% sensitivity and 52% specificity (95% CI 0.604–0.771).

**FIGURE 2:**
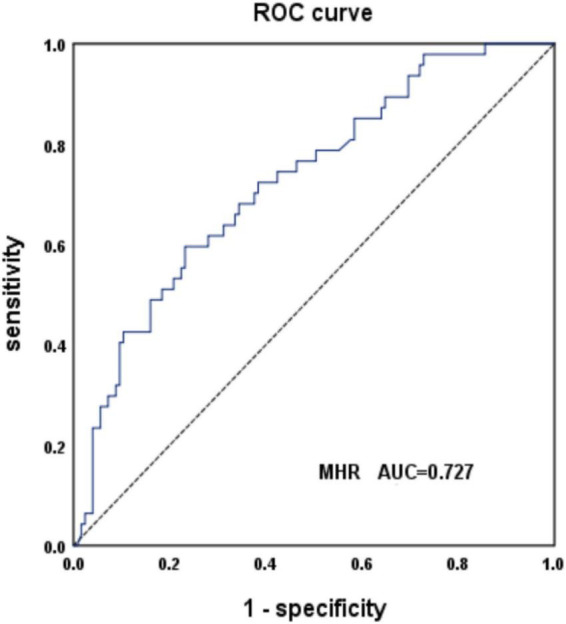
Receiver operating characteristic (ROC) curve analysis of monocyte to high-density lipoprotein ratio (MHR) to predict multiple system atrophy (MSA) and PD.

The AUC of MSA and PD predicted by MHR was 0.727, with a sensitivity of 60% and specificity of 77% (95% CI: 0.644–0.81, *P* < 0.001), when the cut-off value was 0.38 (Figure 2). The data are shown in Table 3.

**TABLE 3:** The predictive efficacy of monocyte to high-density lipoprotein ratio (MHR), neutrophil to lymphocyte ratio (NLR), and red cell distribution width to platelet ratio (RPR) in the diagnosis and differential diagnosis of multiple system atrophy (MSA).

	Indicator	AUC	Sensitivity (%)	Specificity (%)	95% CI	*P*	Cut-off
MSA vs. HC	MHR	0.651	0.750	0.520	0.562–0.740	0.002	0.300
	NLR	0.600	0.320	0.880	0.501–0.699	0.045	2.990
	RPR	0.612	0.810	0.400	0.516–0.708	0.024	0.060
	Combination	0.688	0.770	0.520	0.604–0.771	<0.001	0.220
MSA vs. PD	MHR	0.727	0.600	0.770	0.644–0.810	<0.001	0.380

### Spearman correlation analysis

We analyzed the correlation between the MHR, NLR, and RPR and disease duration in different types of MSA patients. The results suggested that the MHR was significantly and positively correlated with the course of MSA-C (Table 4). The baseline data of different types of MSA are shown in Appendix 1.

**TABLE 4:** Correlation analysis between monocyte to high-density lipoprotein ratio (MHR), neutrophil to lymphocyte ratio (NLR), red cell distribution width to platelet ratio (RPR), and disease course of multiple system atrophy (MSA) patients of different types.

	Disease duration of MSA-C		Disease duration of MSA-P	
	ρ	*P*	ρ	*P*
MHR	0.43	0.025	−0.371	0.107
NLR	0.19	0.342	−0.151	0.526
RPR	0.008	0.97	−0.235	0.319

## Discussion

The etiology of MSA is unclear, and its possible mechanism is related to intracellular oxidative stress and inflammation caused by abnormal aggregation of α-synuclein. A number of studies have demonstrated that central inflammation exists in both MSA models and patients. Furthermore, neuroinflammation accelerates neuronal cell death (Fanciulli and Wenning, 2015). For example, Hoffmann et al. (2019) found in a mouse model of MSA that increased gene expression of proinflammatory cytokines was detected in oligodendrocytes overexpressing α-synuclein. This may be related to the proliferation and activation of myeloid cells caused by oligodendrocyte overexpression of α-synuclein, thus resulting in the proliferation of microglial cells and infiltration of macrophages. Surveys such as those conducted by Yamasaki et al. (2017) showed that cytokines with proinflammatory, anti-inflammatory, and chemotactic effects in the cerebrospinal fluid of patients with MSA were significantly increased, such as IL-6, IL-7, IL-12, IL-13, and GM-CSF. This is similar to the findings of Compta et al. (2019). Studies on autopsy and biopsy of MSA patients have suggested that inflammatory factors increase in brain parenchyma, which may be related to the proliferation of microglia caused by neuron damage and the release of inflammatory factors (Rydbirk et al., 2017; Li et al., 2018). However, is peripheral inflammation also involved in the pathogenesis of MSA? At present, there are few relevant studies and inconsistent views. In, Kaufman et al. (2013) first explored peripheral inflammatory indicators [IL-6, IL-2, C-reactive protein (CRP), and TNF-α] in the serum of MSA patients, and the study confirmed that the levels of IL-6 and TNF-α in MSA patients were significantly higher than those in the control group. In contrast, similar studies conducted by Kim et al. (2019) did not yield the same results. Taken together, the relationship between MSA and peripheral inflammation needs to be further studied.

Monocytes can induce the production of cytokines with proinflammatory effects, such as TNF and IL-10, and have high antigen-presenting activity, participating in activation and phagocytosis in the inflammatory process (Ziegler-Heitbrock, 2007). Moreover, studies have shown that monocyte chemotactic protein (MCP-1), which plays a role in monocyte recruitment, is significantly increased in the cerebrospinal fluid of MSA patients (Magdalinou et al., 2015). HDL is a kind of plasma lipoprotein that can transport cholesterol to reduce lipid accumulation and has anti atherosclerotic effects. It was reported that HDL could inhibit the activation of white blood cells, neutrophils, and monocytes and played an anti-inflammatory role (Murphy et al., 2009). HDL-C has an antioxidant function by inhibiting the deposition of toxic proteins, and can be used as a protective factor for the onset of MSA (Lee et al., 2009). Therefore, there may be a link between the ratio of HDL to monocytes and inflammation. In recent years, studies have shown that the MHR can be used as an inflammatory indicator to predict the mortality of mesenteric artery embolism, the mortality of acute ischemic stroke within 1 month and the recurrence rate of atrial fibrillation after radiofrequency ablation (Bolayir et al., 2018; Chen et al., 2020; Kuvvetli and Avci, 2020). Considering that inflammation plays a significant role in the pathophysiological process and prognosis of synucleinopathy, such as MSA and PD, we explored the relationships among MHR, MSA, and PD. The results showed that, in terms of MHR, the MSA group and PD group were significantly higher than the HC group, which may be related to the fact that both MSA and PD belong to α-synucleinopathy. Studies have shown that inflammation is involved in the pathogenesis of α-synucleinopathy (La Vitola et al., 2021). In addition, the MHR level of MSA was significantly higher than that of PD, suggesting a higher level of inflammation in MSA. In fact, as a Parkinson-plus syndrome, MSA involves multiple systems and has a more complex pathological mechanism than PD. Therefore, the level of inflammatory factors may be more prominent. In most cases, we can also observe that MSA patients have more serious clinical manifestations and worse prognoses than PD patients (Schapira et al., 1992; Benecke et al., 1993).

Neutrophils and lymphocytes are important indicators of inflammation and are linked to the occurrence, progression, and severity of inflammation. Some studies have shown that the secretion of IL-2 and IL-15, which are involved in T lymphocyte maturation, is significantly increased in the serum of patients with MSA. In addition, the numbers of CD3 + T lymphocytes and CD4 + T lymphocytes were increased in the peripheral blood of MSA patients (Csencsits-Smith et al., 2016; Cao et al., 2020). The NLR, the ratio between neutrophils and lymphocytes, is considered as a subclinical inflammatory index in European and American countries and can reflect the intensity of inflammation (Azab et al., 2014). Studies have reported associations between NLR and systemic inflammation in a variety of diseases. It has previously been reported that NLR may reflect the peripheral inflammatory processes associated with Alzheimer’s disease (Rembach et al., 2014). A study on peripheral inflammation in patients with PSP found that NLR was associated with PSP but not with PD, which can be used as a predictor to distinguish PSP from PD (Inci et al., 2020). In our study, there was no statistically significant difference in NLR between the PD group and the control group, which was inconsistent with the research results of Liu et al. (2021). A possible explanation for this might be that the PD population included by Liu is located in Qinghai Province at a high altitude. The environment, especially under low oxygen conditions, is an important factor affecting the occurrence and development of PD. Hypoxia can lead to oxidative stress, increase the proinflammatory process and aggravate neuronal injury (Leston et al., 2021). In addition, the special geographical environment of low oxygen and low air pressure in high-altitude areas will cause disorders of human metabolism and immune function. Studies have shown that there is a statistically significant difference in leukocytes between people living in high- and low-altitude areas for a long time, which is the result of immune system regulation (Alkhaldy et al., 2020). Therefore, the correlation between NLR and PD needs to be further verified by a multicenter study. However, the NLR value of the MSA group was higher than that of the healthy control group (*P* < 0.05), suggesting a correlation between MSA and NLR. MSA may have peripheral inflammation.

Red blood cell distribution width is an indicator of the variability of red cell volume in blood. Studies have shown that inflammatory factors inhibit erythrocyte maturation by reducing the sensitivity of hematopoietic stem cells to erythropoietin (EPO) (Forhecz et al., 2009). Previous studies Solak et al. (2014) revealed a strong positive correlation between RDW and CRP. PLTs are involved in inflammatory processes by mediating leukocyte adhesion and extravasation (Klinger and Jelkmann, 2002; Polak et al., 2020). Thrombocytosis is an indicator of an increased degree of suppurative infection (Tchebiner et al., 2011). In recent years, RPR, as a new inflammatory indicator, has been proven to be a predictive indicator to distinguish hepatic fibrosis from cirrhosis, the severity of cirrhosis, and the 3 months survival rate after thrombectomy for acute cerebral infarction (Chen et al., 2013; Wang et al., 2016; Li et al., 2021). However, there are no relevant studies on RPR in neurodegenerative diseases. The findings of this study suggested that compared with the HC group, the RPR of the MSA group and PD group was higher (*P* < 0.05), while the platelet counts of the MSA group and PD group was lower. Previous studies have found that the activity of enzymes involved in the oxidative respiratory chain in PLTs of PD patients is reduced by half, resulting in decreased ATP production, which may affect platelet growth or proliferation.

This study explored the relationship between MSA and peripheral inflammatory markers (NLR, MHR, and RPR). The results of this study confirmed the correlation between MSA and peripheral inflammation and further provided evidence that peripheral inflammation was involved in the pathogenesis of MSA. However, limitations exist in this study. First, our study was a retrospective study. The time points of blood samples collected in the included population were different. Additionally, our study was a single-center study. The incidence of MSA is relatively low, and the number of MSA patients included is small. Moreover, the diagnosis of MSA is exclusive. However, typical clinical manifestations and pontine “hot cross bun sign” contribute to the diagnosis of MSA. Nevertheless, a considerable number of early stage patients do not show significant clinical characteristics. Specialists with rich clinical experience in motion disorders are also often unable to accurately identify them. Thus, there may be selection bias. In view of the fact that the sensitivity and specificity of the indicators in this study are not very high, it may be necessary to combine with the common autonomic symptoms of multiple system atrophy such as postural hypotension and urinary symptoms in subsequent studies in order to improve the sensitivity and specificity of the testing index. In addition, in subsequent studies, it is also necessary to strictly and accurately control the diagnostic criteria and include adequate sample sizes in prospective and multicenter studies.

## Conclusion

In conclusion, peripheral inflammation is correlated with MSA and is connected with the onset of MSA. The study further confirmed that MSA may be higher than PD at the level of inflammation. In addition, the MHR may be used as an indicator of differential diagnosis between MSA and PD. It has certain clinical value and significance. However, despite the presence of peripheral inflammation in MSA, it is not clear whether inflammation is a trigger or a consequence of MSA. The specific related pathways remain unclear and need to be further explored by basic research.

## Data availability statement

The original contributions presented in this study are included in the article/supplementary material, further inquiries can be directed to the corresponding authors.

## Ethics statement

The studies involving human participants were reviewed and approved by the Second Affiliated Hospital of Nanchang University. The patients/participants provided their written informed consent to participate in this study.

## Author contributions

LJ wrote the manuscript. HB, ZZ, and JH reviewed and revised the manuscript. WH directed the study. All authors contributed to the article and approved the submitted version.

## Appendix

**APPENDIX 1:** Baseline information for multiple system atrophy cerebellar type (MSA-C) and multiple system atrophy-parkinsonism (MSA-P).

	MSA-C (*n* = 27)	MSA-P (*n* = 20)	*Z*/*T*	*P*
Age (years)	60 (54–66)	61 (56–65.75)	–0.356	0.722
Sex (male, %)	18 (63.8%)	72 (57.6%)	0.221	0.638
Smoking (%)	5 (10.6%)	10 (8%)	4.145	0.042
Drinking (%)	7 (14.9%)	12 (9.6%)	0.658	0.417
Hypertension (%)	12 (25.5%)	19 (15.2%)	1.641	0.200
Disease duration (month)	12 (8–24)	24 (8.25–45)	–1.392	0.164
